# Sacral neuromodulation in children and adolescents with chronic constipation refractory to conservative treatment

**DOI:** 10.1007/s00384-016-2604-8

**Published:** 2016-06-13

**Authors:** Aart A. van der Wilt, Bart P. W. van Wunnik, Rosel Sturkenboom, Ingrid J. Han-Geurts, Jarno Melenhorst, Marc A. Benninga, Cor G. M. I. Baeten, Stephanie O. Breukink

**Affiliations:** Department of Surgery, Maastricht University Medical Center, P. Debyelaan 25, 6202 AZ Maastricht, The Netherlands; Department of Pediatric Gastroenterology, Academic Medical Center, Amsterdam, The Netherlands

**Keywords:** Sacral neuromodulation, Constipation, Children, Adolescents

## Abstract

**Purpose:**

Functional constipation in children and adolescents is a common and invalidating condition. In a minority of patients, symptoms persist despite optimal conservative therapy. The aim of this study was to evaluate whether the short-term effects of sacral neuromodulation (SNM) in children and adolescents with constipation are sustained over prolonged period of time.

**Methods:**

Patients aged 10–20 years, with refractory constipation, fulfilling the Rome III criteria, were included in our study. If SNM test treatment showed >50 % improvement in defecation frequency, a permanent stimulator was implanted. Primary outcome measure was defecation frequency during 3 weeks. Secondary endpoints were abdominal pain and Wexner score. To assess sustainability of treatment effect, a survival analysis was performed. Cross-sectional quality of life was assessed using the EQ-5D VAS score.

**Results:**

Thirty girls, mean age 16 (range 10–20), were included. The mean defecation frequency increased from 5.9 (SD 6.5) in 21 days at baseline to 17.4 (SD 11.6) after 3 weeks of test treatment (*p* < 0.001). During test treatment, abdominal pain and Wexner score decreased from 3.6 to 1.5 and 18.6 to 8.5 (*p* < 0.001), respectively. Improvement of symptoms sustained during a median follow-up of 22.1 months (12.2–36.8) in 42.9 % of patients. On a scale from 0 to 100, quality of life was 7 points lower than the norm score (mean 70 vs. 77).

**Conclusion:**

SNM is a therapeutic option for children with chronic constipation not responding to intensive oral and/or laxative therapy, providing benefits that appear to be sustained over prolonged period of time.

## Introduction

Constipation is a common problem in childhood and adolescence, with a prevalence ranging between 0.7 and 29.6 % (median 12 %) [1]. Functional constipation, as defined by the ROME III criteria [2], has significant impact on quality of life, both for the patient as for his or her family [3].

Various modalities have been developed in the treatment of functional constipation, including increase of dietary fiber intake, osmotic and stimulant laxatives, retrograde bowel irrigation, and behavioral therapy, including biofeedback training and pelvic floor physiotherapy [1, 4]. In the majority of patients, constipation can be effectively treated by these conservative treatments [5].

A group of patients exists, however, in whom symptoms persist for many years. In these patients, abdominal pain, most likely due to the low defecation frequency, is severely disabled in their daily activities, such as attending school and participation in sports and social events. For these refractory patients, surgical procedures such as antegrade bowel irrigation by means of a Malone stoma, colectomy, or segmental colonic resection may be considered [6]. Such treatments can, however, incur complications such as small bowel obstruction, chronic diarrhea, fecal incontinence, and abdominal pain; moreover, long-term results are highly variable [7].

Sacral neuromodulation (SNM) was initially developed as a treatment for urinary incontinence and retention in adults [8]. In these series, a subset of patients who suffered from urinary and fecal incontinence experienced improvement in both. In adults, studies of SNM for functional bowel complaints showed a positive effect on idiopathic slow and normal transit constipation resistant to conservative treatment [9]. Our preliminary data suggest that SNM may also be beneficial in young patients aged 10–20 years with refractory constipation [10]. It is unknown, however, whether relief of symptoms is sustained. In this study, we report longer term follow-up results with SNM in children and adolescents with constipation refractory to intensive conservative treatment.

## Patients and methods

A prospective cohort study was conducted in a tertiary referral hospital to assess the efficacy of SNM in adolescents with refractory constipation. All patients between 10 and 20 years of age, who were referred to our center for chronic (>1 year) constipation refractory to conservative treatment, were eligible to be included in this study. Informed consent was obtained from all patients and their parents.

All patients met the criteria for functional constipation, as defined by the Rome III criteria [2]. Demographic data, disease history, and prior treatments were collected from all patients. Severity of symptoms was determined at baseline by means of a 3-week bowel diary, subjective questionnaires, and Wexner constipation scores [11]. A bowel diary was filled in during a period of optimal conservative treatment, consisting of a combination of different oral and/ or rectal laxatives. Patients were asked to report the frequency of defecation, presence of straining, episodes being unable to evacuate, urge, painful defecation, size of stool, abdominal pain, and absence from school due to constipation-related complaints.

The Wexner score is a composite score ranging from 0 to 30 based on eight domains including frequency, difficulty to evacuate, completeness, painfulness, duration (time in lavatory), successfulness, and need for assistance in defecation, as well as history (duration of symptoms) [11].

Further diagnostic tests included defecography to assess possible outlet obstruction, anal ultrasound, anorectal manometry to exclude Hirschsprung’s disease, colonic transit time measurement using radio-opaque markers, and MRI of the lumbar spine.

### Operative details

The operative technique for SNM has been described in detail elsewhere [12]. Briefly, patients had a tined lead (Medtronic Interstim model 3889) placed in order to perform a test treatment of sacral neuromodulation. This procedure took place under general anesthesia with antibiotic prophylaxis and was performed by an experienced surgeon. An X-ray was used to determine the location of S3 and to place a needle in the foramen. Correct position of the needle was confirmed by Bellow’s contraction. Finally, the electrode was placed in position and connected to the external stimulator. This was followed by a 3–5-week test period to assess treatment benefit and decide on implantation of the permanent stimulator. To qualify for a permanent device, defecation frequency had to be restored to a frequency of minimally three times per week. The permanent implantable stimulator (Medtronic Interstim model 3058) was implanted under local anesthesia. It was placed in a subcutaneous gluteal pocket under antibiotic prophylaxis. Initial stimulation settings were identical to those during the testing phase. These stimulation settings are identical to those used for adults. Initially, a pulsewidth of 210 μs and a frequency of 16 Hz were used. In the case of unpleasant sensations or suboptimal treatment effect, settings could be changed from 120 to 330 μs and 10 to 21 Hz, respectively.

### Assessment and follow-up

Patients were followed up at 1, 3, 6, 12, and 24 months after implantation of the pacemaker. Prior to each follow-up moment, a 3-week bowel diary was completed, and questionnaires and Wexner constipation scores were recorded. Additional use of laxatives, recurrence of symptoms, or technical failure with or without reoperation was recorded. The primary endpoint was the frequency of defecation as recorded in the 3-week bowel diary. Treatment was considered successful when defecation frequency was at least three times per week.

Secondary outcome parameters were change in Wexner constipation score, presence of abdominal pain, pain at defecation, straining, feeling of urge, incomplete evacuation, general comfort, and quality of life. Abdominal pain, pain at defecation, straining, feeling of urge, and incomplete evacuation were assessed using a 5-point scale, ranging from 0 “never,” 1 “rarely,” 2 “sometimes,” 3 “most of the times,” to 4 “always.” Quality of life was assessed by means of the EQ-5D VAS [13]. Recurrence of constipation was defined as a defecation frequency less than three times per week or the need for use of laxatives or bowel lavage to control symptoms.

### Statistical analysis

Data are presented as mean (SD) or median (range) for continuous variables, and count (percentage) for categorical variables. Changes over time in severity of symptoms were tested for statistical significance using paired *t* test and Wilcoxon signed ranks test. A time-to-event analysis was conducted using Kaplan-Meier survival analysis. Event was defined as a remission to a defecation frequency of less than three times per week, with or without lead or pocket revision. Analyses were conducted using SPSS. A *p* value of 0.05 was considered statistically significant.

## Results

Thirty patients with refractory constipation were referred to our center between February 2009 and December 2011. Table 1 shows the patient characteristics and the progress of abdominal pain and defecation frequency over time. All 30 patients underwent a test stimulation of SNM. In three of these patients, test stimulation was ineffective and the electrode was removed. All remaining 27 patients had a stimulator implanted and were available for follow-up. Median follow-up was 22.1 months (12.2–36.8).

**Table 1:** Baseline characteristics and follow-up

Patient	Age	Duration of symptoms (years)	Admissions	CTT (h)	Manometry RAIR	Abdominal pain baseline	Abdominal pain test	Abdominal pain 1 year	Def bl/3 weeks	Def test/3 weeks	QoL EQ5D-VAS
1	18	18	m	60	np	4	3	2	1	6	m
2	16	6	2	np	np	3	2	2	6	6	43
3	15	9	11	np	RAIR+	4	2	3	3	15	93
4	14	9	10	144	np	4	2	2	1	10	65
5	19	18	20	148	RAIR+	4	2	2	3	12	68
6	17	17	50	148	RAIR+	4	1	1	9	32	54
7	17	16	20	92	RAIR+	4	2	1	1	9	57
8	15	14	50	50	RAIR+	4	1	3	2	3	m
9	13	1	10	50	RAIR+	4	2	2	6	17	91
10	10	5	12	80	RAIR+	3	2	0	8	9	22
11	14	4	m	np	RAIR+	3	3	x	3	0	x
12	19	10	2	96	RAIR+	4	0	2	6	54	91
13	14	14	6	np	RAIR+	4	1	m	21	8	69
14	18	16	3	62	RAIR+	4	2	2	1	21	71
15	17	5	3	38	RAIR+	3	1	1	9	20	75
16	14	6	3	np	np	3	1	1	9	19	70
17	13	13	30	144	RAIR+	4	3	m	3	15	m
18	18	2	12	np	RAIR+	4	1	2	20	27	60
19	16	2	1	48	RAIR+	4	0	1	1	24	m
20	15	3	7	136	RAIR+	2	0	1	8	10	71
21	16	3	4	132	RAIR+	3	0	m	3	20	80
22	16	1	7	67	RAIR -	3	2	x	1	1	x
23	16	3	9	96	RAIR+	4	4	m	21	20	m
24	16	10	3	74	RAIR+	4	4	1	0	4	91
25	16	1	6	86	np	4	1	m	0	15	79
26	16	8	2	120	RAIR+	3	0	0	0	39	89
27	15	15	25	19	RAIR+	4	0	m	3	27	59
28	15	8	15	139	RAIR+	3	3	x	0	0	x
29	15	1	3	np	np	4	3	m	0	9	m
30	16	2	2	np	np	2	1	2	2	8	m

Abdominal pain: 4 always, 3 most of the tome, 2 sometimes, 1 rarely, 0 never

*np* not performed; *m* missing; *x* electrode removed, no follow-up data available; *Def bl* defecation frequency at baseline; *Def test* defecation frequency during test phase; *RAIR* recto-anal inhibition reflex: + intact, − no RAIR

### Baseline characteristics

All patients were female with a mean age of 16 years (range 10–20) at the time of the test stimulation. Mean duration of complaints was 8.1 years (range 1–18). At the time of presentation in our outpatient clinic, all patients had been treated extensively with various conservative treatments for at least 1 year, under supervision of a referral center. All patients used laxatives, including polyethylene glycol, Metamucil, lactulose, bisacodyl, and magnesium oxide, or a combination of these, with or without retrograde bowel irrigation. Duration of laxative use ranged from 1 to 17 years (mean 5.9 years). One patient had a Malone stoma for antegrade bowel irrigation. All patients had been admitted multiple times to hospital for either oral and/or rectal lavage (median 7, range 2–50).

Colonic transit time was assessed in 22 of 30 patients and showed a mean colonic transit time of 92.3 h. Fifteen patients had colonic transit times exceeding 62 h (Table 1). Lumbar spine MRI was performed in 22 of 30 patients and revealed no spinal abnormalities. In 23 of 30 patients, an anorectal manometry was performed, which showed an abnormal result in one patient, in whom the anorectal inhibition reflex could not be elicited (Table 1). In the latter patient, a rectal suction biopsy was performed which showed normal ganglion cells. Results of defecography were available for 21 patients. In five, there was a minimal sign of rectocele (grade 1, smaller than 4 cm), and one patient showed a rectocele, enterocele, and intussusception. Six patients had no evacuation of the contrast at all, and in eight patients, evacuation was incomplete. Twelve patients underwent anal ultrasound. Two of them showed a defect of approximately 30–45° in the internal and the external sphincter, respectively. For one, the defect was possibly the result of sexual abuse. In the remaining 10 patients, no abnormalities were found on anal ultrasound.

### Defecation frequency

At baseline, the mean defecation frequency during a 3-week period was 5.9 (SD 6.5) or 1.96 times per week. During the test phase, the mean defecation frequency increased to 17.4 (SD 11.6) per 3 weeks or 5.8 times per week. During follow-up, the mean defecation frequency remained stable at this level. At each time at follow-up, the defecation frequency was significantly higher when compared to baseline defecation frequency (*p* < 0.001).

### Secondary outcome parameters

**Fig. 1:**
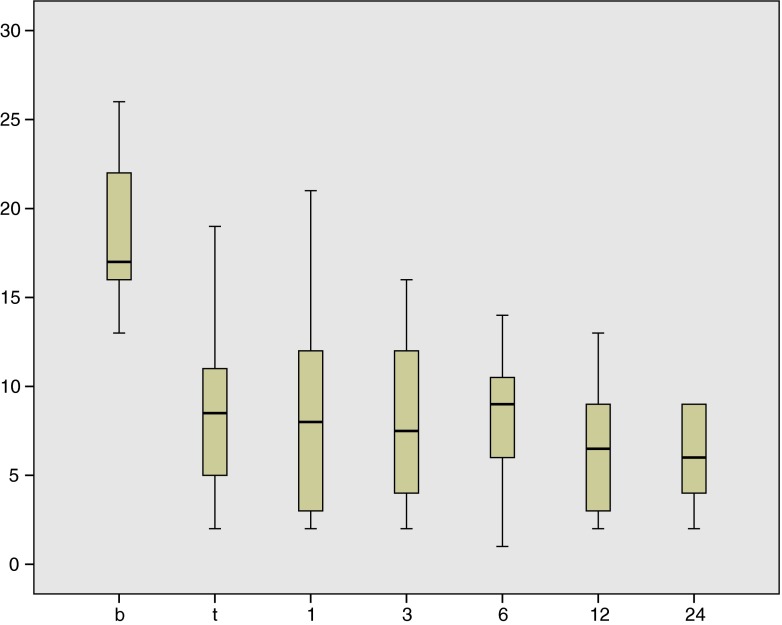
Box plot of Wexner scores at baseline (*b*), during test phase (*t*), and at 1, 3, 6, 12, and 24 months follow-up, respectively. *n* = 30/30 (baseline), *n* = 30/30 (test), *n* = 27/27 (*1*), *n* = 24/27 (*3*), *n* = 25/27 (*6*), *n* = 20/27 (*12*), and *n* = 10/25 (*24*). *x*-axis: time. *y*-axis: Wexner score

The mean Wexner score decreased from 18.6 (SD 8.5) at baseline to 8.2 (SD 8.3) during test phase and remained stable during follow-up (Fig. 1). At each follow-up visit, the mean Wexner score was significantly lower as compared to baseline (*p* < 0.001). The abdominal pain score decreased from mean 3.62 at baseline to 1.53 at 1 year follow-up (*p* < 0.001). At baseline, two patients reported to have abdominal pain some of the time, while the other patients reported to have abdominal pain most of the time (9) or always (19). At 1 year follow-up, two patients still had abdominal pain most of the time, nine sometimes, seven rarely, and two never. Accompanying symptoms such as pain at defecation, straining, and incomplete evacuation showed a comparable decrease, while the times of feeling of urge increased. The EQ5D VAS score was 69.90 (SD 17.96) at a median follow-up of 12 months, which is lower than the norm score for healthy Dutch females aged 15–19 (mean 76.73, SD 12.58) (Table 1) [13].

### Complications and revisions

A total number of 15 revisions were performed in 12 patients. Reasons for revision were the following: recurrence of symptoms in six patients, a lead revision was performed; pain at the site of the stimulator in five patients, the position of the stimulator was revised; and infection of the electrode in one patient, the electrode was removed and a new test was performed after 4 weeks. No other, more serious adverse events associated with the surgical procedure were observed. At the end of follow-up, 21 of 27 patients were still on SNM therapy.

Fifteen of 27 patients failed on SNM, six of these patients underwent a total colectomy and subsequently had the stimulator removed. The remaining nine patients combined SNM with laxatives and/or bowel irrigation.

### Survival analysis

**Fig. 2:**
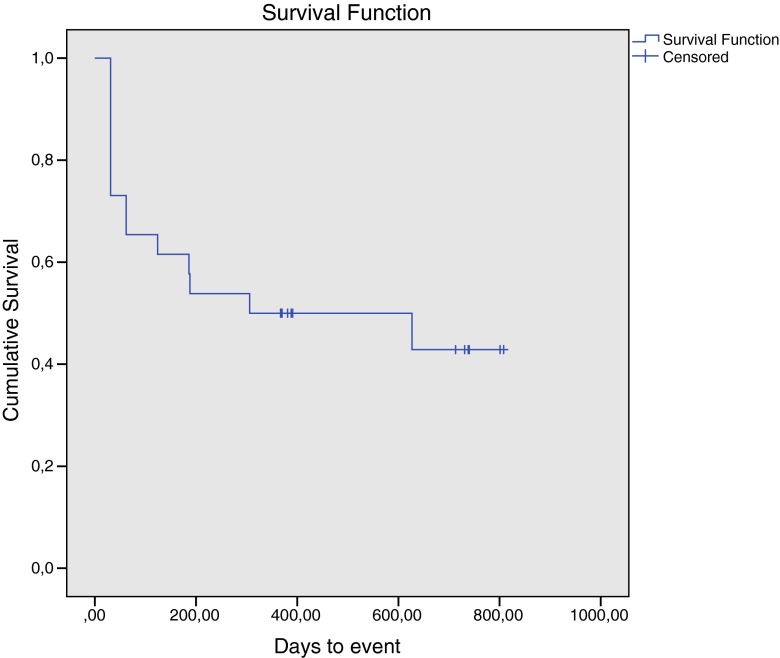
Kaplan-Meier survival curve, showing recurrence of constipation symptoms over time. Censored observations represent end of follow-up. End-point is recurrence of symptoms, with or without reoperation. *x*-axis: days to event. *y*-axis: cumulative survival

Figure 2 shows the results of the time-to-event analysis, where an event is defined as a decrease of defecation frequency below three times per week, with or without revision. The figure shows that in this population of patients with chronic constipation, refractory to conservative treatment, the 2-year recurrence-free survival was approximately 42.9 %. These events mainly occurred in the first year after treatment, as after 6 and 12 months, the recurrence-free survival was 58 and 50 %, respectively.

## Discussion

This study shows that in some patients, SNM has a long-lasting beneficial effect on defecation frequency and accompanying symptoms such as abdominal pain and difficulties with evacuation in adolescent patients with severe constipation resistant to conservative treatment.

Sacral nerve modulation in our cohort of children and adolescents with therapy-resistant constipation shows similar efficacy and safety rates as in adults with refractory constipation [9]. Improvement in defecation-related parameters has been reported in adults, in terms of defecation frequency, abdominal pain, and use of laxatives. Many different outcome parameters are used however, which makes adequate comparison difficult, and this probably also results in the wide range of success rates (42–100 %) [14, 15]. In the prospective study by Kamm et al., the indication to proceed to permanent implantation was an improvement of at least 50 % in defecation frequency or a frequency of three or more per week as well. This resulted in a success rate of 63 % at a median follow-up of 28 months [9]. The higher success rates reported of SNM for constipation in adults compared to the current population is possibly a result from the severity of symptoms these adolescents experience. Moreover, this finding contributes to the suggestion of different etiologies of constipation for children and adults [1].

A recent study in children with fecal and urinary incontinence and constipation reported that six of 11 patients with a pre-SNM cecostomy tube no longer required an antegrade bowel regimen as they had voluntary bowel movements [16]. Furthermore, ten of 11 patients (91 %) no longer required anticholinergic medications for bladder overactivity after receiving SNM. Since the short-term follow-up in this small heterogeneous group of patients, longer follow-up studies are required to identify longer-term effects of SNM for patients with bowel and bladder dysfunction and potentially identify subgroups of patients that are more likely to respond to sacral neuromodulation. Our study shows that the 2-year recurrence-free survival was approximately 42.9 %, without major complications. Based on these results, we decided not to remove the permanent stimulator in these successfully treated patients. Data are lacking to estimate after which time it is possible to switch off and remove the stimulator [9, 16].

Of the 21 patients who underwent defecography, only 1 did not show some degree of evacuatory dysfunction. Knowles et al. suggested that evacuatory dysfunction should not be an exclusion criterion for SNM [17]. It might even lead to sensory improvement in patients suffering from rectal hyposensitivity. Unfortunately, follow-up data of manometry to assess dyssynergia and rectal sensitivity are not available for the cohort of patients in this trial.

In 15 out of 22 patients in whom colonic transit time was assessed at baseline, colonic transit appeared to be delayed. The increase in defecation frequency suggests that colonic transit time improves after SNM. Clarke et al. concluded that colonic transit time was significantly shorter after interferential therapy (IFT) compared to sham [18]. IFT, a form of transcutaneous electrical nerve stimulation (TENS) [19, 20], is similar to SNM as both techniques aim to modulate neural activity by electrical stimulation. However, IFT is even less invasive as it makes use of surface electrodes and transcutaneous stimulation instead of the percutaneous implantation of an electrode, which is connected to an implantable stimulator. Indeed, children with slow transit constipation, who failed conventional therapy, were effectively treated with TENS [19, 20]. More importantly, after daily treatment for 1–6 months, colonic function improved for several years and some children were even able to withdraw medication. Large randomized controlled trials are required to compare both neuromodulation techniques in both children and adults with refractory constipation.

The extensive diagnostic workup prior to the test period was performed in an attempt to identify possible prognostic factors for success regarding SNM. Unfortunately, the number of patients in this study is too small to draw any conclusions in this regard.

The high rate of revisions in this population is the major drawback of this surgical intervention. In accordance with the other pediatric study, in approximately half of the cases, the indication for revision was pain in the area of the implanted stimulator [16]. This is despite the fact that we already implanted the smallest device (Medtronic Interstim2, model 3889). The other revisions were because of recurrence of symptoms. Important to note is that the present population is very young and active, which may partially explain the higher lead revision rate when compared to adults [21]. Another factor that may be of influence is the fact that these young patients are still growing, affecting lead position [22]. The majority of relapses that could not be resolved by revision occurred during the first year after implantation. Possible explanations for such relapses include gradual adjustment of the pelvic floor and colon to the new stimulation or weaning of an initial placebo effect or other psychological phenomenon.

For fecal incontinence, SNM appeared to be cost-effective [10, 23]. For constipation, these analyses have not been performed yet. The high costs associated with the technique make funding more problematic, which probably contributes to the fact that this treatment is not commonly used. The direct costs from the electrode and the stimulator are approximately 8000 euros [24]. Childhood constipation is a common problem and brings a great economic burden to the public health system [25]. The study by Liem et al. reported that in the USA, the yearly costs of children with constipation were three times higher than those of children without constipation [26]. So, adequate treatment of this condition potentially leads to a major reduction in costs due to frequent admissions. For a treatment to be cost-effective, the effect on symptoms and quality of life needs to be taken into account. Unfortunately, from the patients in this study, no data on quality of life before treatment are available. However, in general, the quality of life seems to be impaired in children with constipation [27]. Therefore, it is promising to see that after SNM, quality of life was only 6 points lower than in the general population (on a scale from 0 to 100), especially considering the fact that this group at baseline suffered from the worst complaints in the spectrum.

A major limitation of our study is its observational, noncomparative nature. Causally attributing the improvement in symptoms that we observed to the SNM therefore critically hinges on the assumption that, in the absence of treatment, no such recovery would have occurred. In a survey conducted among residents of Olmsted County, MN, aged 30–64 years, Talley et al. (1992) studied the onset and disappearance of symptoms consistent with functional gastrointestinal disorders, including constipation. They found that 89 % of the population surveyed had no change in their symptoms during a period of 12–20 months, suggesting a relative stability in this patient population [28]. Similarly, we cannot exclude the possibility that at least part of the observed improvement may result from a nonspecific placebo effect [29]. To explore these issues further, a randomized, double-blind trial is currently prepared in our center, comparing SNM with sham SNM in children and adolescents with chronic constipation refractory to conservative treatment. A debate among healthcare professionals, patients, and commissioning organizations on the question whether such a study would be appropriate and ethically acceptable is necessary. In such a study, attention should be addressed to the fact that studies on SNM with an observational design encounter the problem that patients who do not benefit from the treatment and proceed to other treatments are lost to follow-up and therefore in the long-term selection takes place. A recently performed review of the literature clearly stated that although SNM appears to be an effective treatment for constipation, larger studies with longer follow-up are needed [15]. An important issue would be to explain heterogeneity in treatment response, allowing for better counseling of patients or patient selection. Clearly, this would require numbers of patients usually not enrolled in single-center studies. To allow for pooled analysis of data from multiple studies in the future, standardization in definitions, assessments, and reporting is vitally important.

In conclusion, for this group of adolescent patients with severe complaints of constipation resistant to conservative treatment, SNM is an effective treatment with beneficial effect on defecation frequency and abdominal pain.
